# Assessment of video see-through smart glasses for augmented reality to support technicians during milking machine maintenance

**DOI:** 10.1038/s41598-022-20154-2

**Published:** 2022-09-21

**Authors:** Gabriele Sara, Giuseppe Todde, Maria Caria

**Affiliations:** Department of Agricultural Sciences, University of Sassari, Viale Italia 39A, 07100 Sassari, Italy

**Keywords:** Mechanical engineering, Computer science, Software

## Abstract

Smart glasses for augmented reality are digital technology under investigation in the agricultural sector. The potential of augmented reality was underlined, in some scientific contributions, as a support tool for farmers' activities and for the decision-making process. One of the most practical applications studied for augmented reality was in maintenance operations, where the use of smart glasses showed high capability. This work focuses on the evaluation of the performance and applicability of smart glasses with a video see-through display system and testing the device's available functions in agricultural frameworks. In addition, an augmented assistance scenario describing the main steps involved in the functioning of the maintenance operation was developed for milking machine inspection. The audio–video quality, battery life, detection capabilities of markers, and voice control interaction system were evaluated. The results showed the capabilities of smart glasses to reach augmented information from a long distance in a short time interval and to transmit audio and video with a high level of detail, allowing discrimination of small objects during remote assistance with reduced delay. The built maintenance scenario represents an example of augmented reality digital assistance application in the inspection and maintenance of the milking machine. The device performance and the proposed maintenance scenario underline the potential that augmented reality could have in the agricultural sector to assist and guide both farmers and technicians to timely problem solving. This solution fits into the agriculture 4.0 perspective, which is increasingly focused on digital transformation to improve farms’ efficiency and sustainability.

## Introduction

Over the last few years, interest in smart farming, precision agriculture, precision livestock farming, and agriculture 4.0^1–3^ has greatly increased. All these terms have been related to different and even complex technologies and to information, digital and robotic technologies, which have been increasingly studied for use in the agricultural sector. The main objectives of introducing this type of technology are to improve farm production in terms of crop or livestock yield, streamline the input use (fertilizer, pesticides, etc.) and reduce waste during the whole production process, leading to more sustainable agriculture^4–6^. Hence, the spread of digital technologies could bring economic, social, and environmental benefits to the agricultural and livestock farming sector in relation to internet access, representing the most critical factor for digital technology diffusion^7,8^. Many new smart technologies have been studied and used to support and simplify farmers' work, such as autoguidance equipment, variable rate technology, and automatic milking systems. Moreover, farm size and education^9^ are influential factors with a large effect on digital farming technology adoption.

Smart glasses (SG) for augmented reality (AR) are a digital technology under investigation in the agricultural sector, where potential applications are underlined as a support tool for farmers' activities and the decision-making process^10–12^. Furthermore, the popularity of AR technologies will grow in the coming years due to improvements in the evolution of cloud computing technologies that will strengthen the power of AR experiences^13^. Possible applications of AR have been found in the maintenance^14^ and manufacturing fields^15^, where AR showed high potential to enhance operator performance. AR-overlayed 3D information may replace paper-based or 2D digital instructions in the assembly/disassembly industrial sector as the use of AR technology was observed to require less time and workload for assembly operators^16^, reducing the error rate^17^. SG have been defined as a wearable computer, despite their lower computational power, and are designed to provide AR experiences through specific applications and visualization systems where virtual or digital contents are projected^18^. The central concept of AR systems concerns overlaying computer-generated contents over the real environment, hence augmenting the physical object with digital information^19^. SG technologies for AR can be considered an emerging technology, even more so in the agricultural sector, for which there are no customized solutions. In addition, AR content authoring is the main obstacle to AR spreading, since AR content creation “requires high effort” and cannot be done “by shop-floor employees”^20^. SG can be distinguished by features, principally for AR element visualization systems (optical see-through, video see-through, and retinal display), the available interaction method (specific button and touchpad placed on the device, external joypad, voice control), and the tracking module integrated into the AR device as well as other characteristics (operating system, processor, functionality, battery life, and framework design)^21–23^.

The tracking module is considered the central system of AR technology, allowing for the identification of the user, device, and environment positions and orientation, anchoring the virtual contents on the physical objects and rendering combined digital and real elements on the AR device display^24^. The tracking system can be supported by different sensors (accelerometer, magnetometer, gyroscope, video camera, RFID, GPS, etc.) in relation to which it is possible to distinguish the technique used by the AR device in vision-based or sensor-based methods. The former case concerns the use of markers (Template, DataMatrix, QR Code) or features of the environment, in contrast with the other cases that use inertial, magnetic, electromagnetic, or ultrasonic sensors to measure and pose the information^14^. Although sensor-based methods have been considered fast and robust, marker-based tracking modules are preferred when high accuracy during position estimation is needed, e.g., in the medical sector^25^. This remains true until the precision and accuracy of the GPS receiver of SG are improved for better AR content positioning, especially for outdoor applications^10^. Moreover, the use of QR code-based techniques have been studied in depth and applied to AR and SG case studies, representing one of the easiest methods to detect and overly augment information^21,22,24^.

The maintenance operations represent one of the main use cases of the AR technologies, where the use and application of AR devices to support operators have been widely studied. In the agricultural sector, the maintenance operations of machines have a high impact on farm management costs, both in terms of time and economical resources, which tend to increase with the machine's hours of use and with the increase in machine fleets^26,27^. Using the AR and SG technologies could reduce the economic impact of machine maintenance operations burdening the farmer. However, AR and SG devices have some issues and challenges to be addressed before their diffusion into the agricultural sector^12,28^. In fact, during maintenance operations the high level of audio and video quality transmission is a key factor for a profitable remote assistance. In addition, the complex pandemic situation caused by the SARS-CoV-2 virus poses new challenges to assist in the field. In this context, guided or remote assistance supported by the SG for AR could play a strategic role in enabling farm workers to solve problems that arise, especially with the machines, to assure their regular maintenance.

The main features of the SG (visualization system, interaction system, and tracking method) could influence user experience and farmer performance using the AR device while working. For this reason, it is important to evaluate and outline the differences among how different SG devices perform in an agricultural context. In fact, the agricultural environment is characterized by diverse operating situations, that may vary throughout the day. The operator performs on-farm activities in environments with different noise levels, lighting conditions (indoor, outdoor), and working space (e.g., milking parlor, barn, open field). Hence, considering the features that characterize SG, the visualization system represents one of the main discriminating characteristics, where, in previous studies, optical display systems were tested in an agricultural context^11,12^. This work focuses on the study and evaluation of the performances and applicability of an SG with a video see-through system, testing the available functions for agricultural frameworks. In fact, this type of visualization system, is considered to have a good visibility of bright and dark virtual contents in different backgrounds (bright and darks) and thus suitable for both indoor and outdoor use^29,30^. Moreover, considering the future employment of SG for AR in the maintenance of the agricultural machines to assist farmers in the field, an AR assistance scenario, which describes the main steps involved in the functioning of the maintenance operation through SG, was developed for milking machine inspection.

## Materials and methods

In this study, a wearable device has been adopted to facilitate maintenance operations. In fact, to accomplish these activities, the operator needs to follow specific procedures that require the use of both hands (e.g., connecting sensors, machine components inspection, etc.), for this reason, handheld devices were not considered in this study. Specifically, the Vuzix® M400 Smart Glasses (M400), produced by the Vuzix Corporation (25 Hendrix Road West Henrietta, NY 14586, USA), were adopted. The M400 is an AR viewer designed for harsh environments (certified IP67), composed of three main components: smart viewer, power bank, and mounting frame. The optical system of the M400 is a video see-through display; on the side of the smart viewer, there is a navigation system that allows interaction with the user interface. There are two types of tangible navigation systems, the classical one, composed of three keys (up, down, enter) and then the touchpad. In addition, there is another navigation system controlled by the voice that allows interaction with the M400 with a preregistered voice command. The power bank is equipped with three LED indicating the status of the battery. In Table 1, the technical specifications of the SG used are stated.

**Table 1:** Main technical specifications of Vuzix M400 Smart Glasses (as reported by the company).

Item	Technical features
Processor	8 Core 2.52 GHz Qualcomm XR1
Flash memory	64 GBytes
Operating System (OS)	Android 8.1
Display	Occluded OLED, 24-bit color, 640 × 360
Field of view	16.8 degrees (5 inches)
Sensors	Gyroscope-accelerometer-magnetometer (3 axis)
Connectivity	GPS, Wi-Fi, Bluetooth, USB
Camera	12.8 Megapixel, 30 fps
Battery	Lithium polymer 1000 mAh (power bank)
Battery life	2–12 h
Controller input	Touchpad, 3 buttons, voice command

Tests were performed in the laboratory of the Agricultural Sciences Department of the University of Sassari. The trials involved evaluating the device’s performance using the available functionalities and applications in controlled light conditions that replicate the milking facilities environment. Specifically, the tests evaluated based on the audio–video quality transmitted and received from the M400, the battery life, the detection of AR markers (QR codes), and, finally, the voice control interaction system. Moreover, a milking machine maintenance framework was built through a specific application to support technicians and farmers during maintenance procedures. The performance tests were designed considering the expected end-use case and thus the maintenance procedures of the milking machine. Thus, getting information on scanning modes allows us to understand where to get the augmented information from, while evaluating the audio–video quality and transmission delay allows understand if real-time assistance in the field is feasible.

All the procedures of this research were carried out in accordance with relevant guidelines and regulations. Informed consent was obtained from all the participating subjects. Moreover, based on previous research protocols the ATS Sardegna ethical committee reported that, when no health care investigators interact within the procedures of the study, the adopted protocol does not require an opinion from the ethics committee.

### QR codes tests

**Figure 1:**
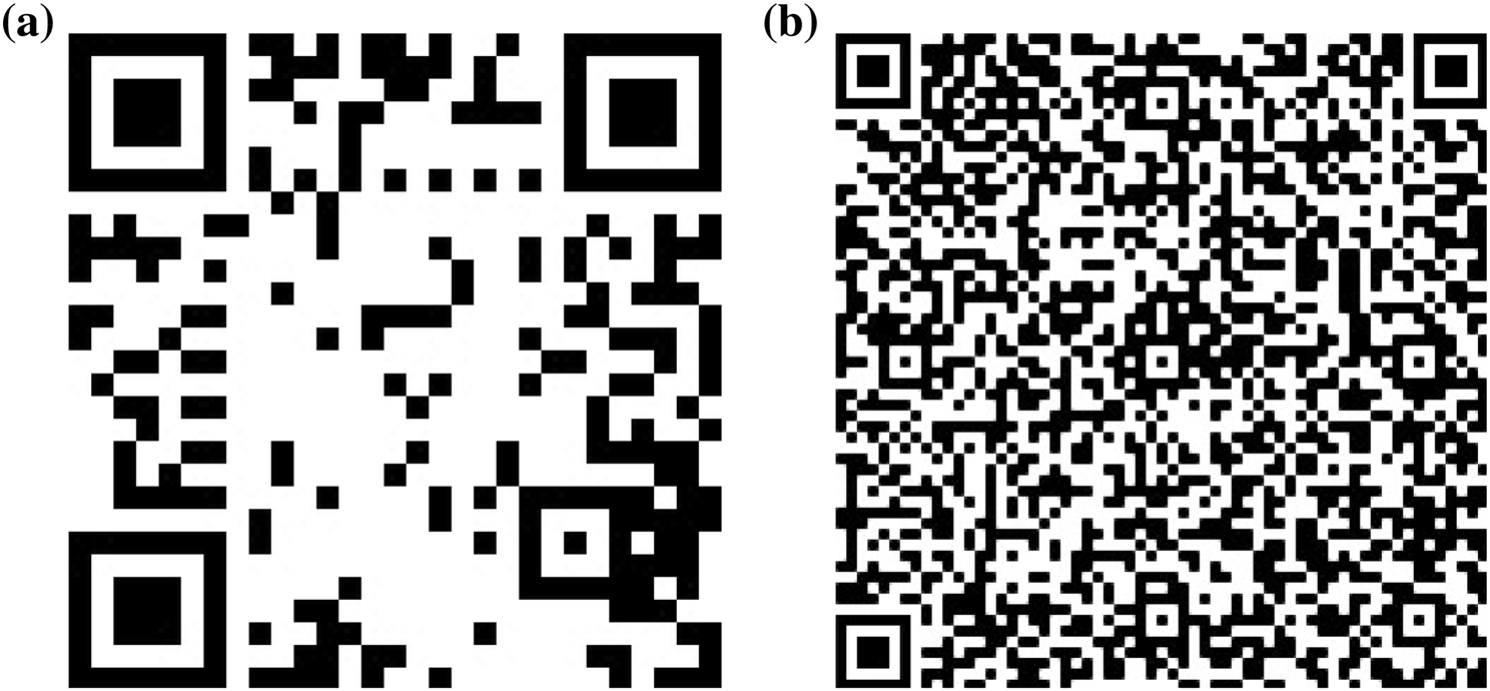
QR codes used during the trial: Version 3 (V3) with 29 modules (**a**); Version 11 (V11) with 61 modules (**b**).

The scanner app was used to evaluate the performance of the M400 (time and distance) with respect to detecting markers that allow us to merge the AR contents onto the real environment. In this study, the QR code was used as a marker due to its capability to store a great amount of information into it, generating up to 40 versions in relation to the encoded information^31^. For the trials, specific farm data were encoded in two different QR code versions. In the first QR code (Fig. 1a), the URL linked to the farm information was encoded. This QR code was a Version 3 (V3) composed of 29 × 29 modules (black or white squares in the QR code). In the second QR code (Fig. 1b), the farm information was directly encoded in text format inside the code, resulting in a Version 11 (V11) composed of 61 × 61 modules.

Using both QR code versions (V3, V11), the minimum and maximum scanning distances were measured from the M400 camera. QR codes with increasing printed size, ranging from 15 mm per side to 200 mm per side, were adopted. To assess the scanning time, i.e., the required time to display the farm information on the SG, three QR codes of different printed sizes (35 mm, 40 mm, 75 mm per side) were tested. The scanning time represents the time frame from the activation of the scanner app to the visualization of the associated information. The codes were placed on the wall at the wearer’s eye level and scanned at 0.4 m. The scanning tests were performed until the end of the battery and repeated for two charging cycles, collecting a total of 2254 records. This test allows us to investigate whether the size and type of QR code affects the time and distance of augmented information gathering. QR codes with high module density can be efficiently used to provide textual augmented information quickly. In fact, encoding a high number of bits (digits) inside the QR code requires a longer time frame but the information will be directly decoded by the SG’s camera.

### Audio video quality transmission test

Two tests were performed to evaluate the audio–video quality contents from the SG to a remote device (PC) using a voice over internet protocol (VoIP) remote call. The first test concerned lag time detection to assess the time needed to transmit audio videos between M400 and laptops. This trial was performed using two different applications: Vuzix® Remote Assist application (App1) and Brochesia® B View application (App2). Both apps are downloadable from the Vuzix App Store and allow to connect field workers, provided with a mobile device (smartphone, smart glasses), to a remote expert (web dashboard) for real-time audio/video cooperation. Moreover, to fully use both applications, subscription is required after the trial version. The lag time of the video and the audio transmitted from the M400 to the laptop were registered by synchronizing the clocks of the two operators and recording the emission and receiving time of a fixed signal for audio and a programmed position for video.

The second test concerns the evaluation of the observable level of detail of the transmitted image through SG. This trial will highlight whether, in a remote video call, the farmer wearing SG would be able to show to a remote operator specific components of a milking machine undergoing maintenance. It was performed using a standard Snellen chart, composed by eleven lines with decreasing size letters blocks. The printed chart was scanned with SG in a 0.5 m distance, and the receiving operator was reading the transmitted chart image on the laptop’s 16-inch screen. The error rate was measured and related to the decreasing size of the characters^32^.

During the trials, the SG and the laptop were connected through App1 and App2 using the University’s Wi-Fi connection (20 Mbps upload/download on average). The tests were carried out with two different video quality resolutions (VGA, 640 × 480 pixels; HD, 1280 × 720 pixels) selectable in the SG application settings and with a Frames per second of 30 fps. For each resolution, the lag time was measured 20 times, whereas the Snellen chart test was performed by two operators. Last, during these trials, the status of the battery in a continuous audio–video transmission was monitored.

### Voice control test

The voice commands were tested to evaluate the noise levels, reachable in different agricultural contexts, that might influence the M400 to recognize voice inputs. The available speech commands were tested at growing levels of noise starting from 65 to 85 dB and in a silent environment (40–50 dB) as a control. The commands tested were 39 voice controls encoded in the device, e.g., “Hello Vuzix”, “Go back”, “Move/scroll down”, “Scroll left”, “Go up”, “Open”, “Select this”, etc. The test was performed by four operators, and the sound pressure levels were monitored using a Trotec SL4 (Trotec GmbH) class 2 sound level meter. During the tests, the voice commands were considered unrecognized after three failed repetitions with an increasing tone of voice.

### Assistance scenario for the farmer

In this work, an assistance scenario was designed through an AR application developed by Brochesia^®^ (BStep). This software application allows building workflows to guide the operator during maintenance activity with punctual augmented information using SG.

The assistance scenario was the measurement of pulsation characteristics related to the mechanical test from milking machines. These measurements commonly involve all the milking units of the milking machine. The workflow was developed according to the International Organization for Standardization (ISO) 6690:2007 and 3918:2007 standards^33,34^, considering the dry test. The assistance procedures were associated with a QR code placed on the milking machine and available through the scanning function of the SG. The workflow for the milking machine inspection process was tested on the milking system in the laboratory of the Agricultural Sciences Department of the University of Sassari (Italy).

### Statistical analysis

Descriptive statistics (arithmetic average, standard deviation, minimum and maximum values) were assessed and are reported for the QR code, the lag time and the voice control tests. Statistical analysis was carried out by performing a Kruskal–Wallis rank sum test for the scanning time and audio–video quality transmission data (non-normal data distribution) and a multiple comparison after the Kruskal–Wallis test (*P* < 0.05). The analyses were performed with R Studio software (version: 4.0.5).

## Results and discussion

### Performance and functionality tests

**Figure 2:**
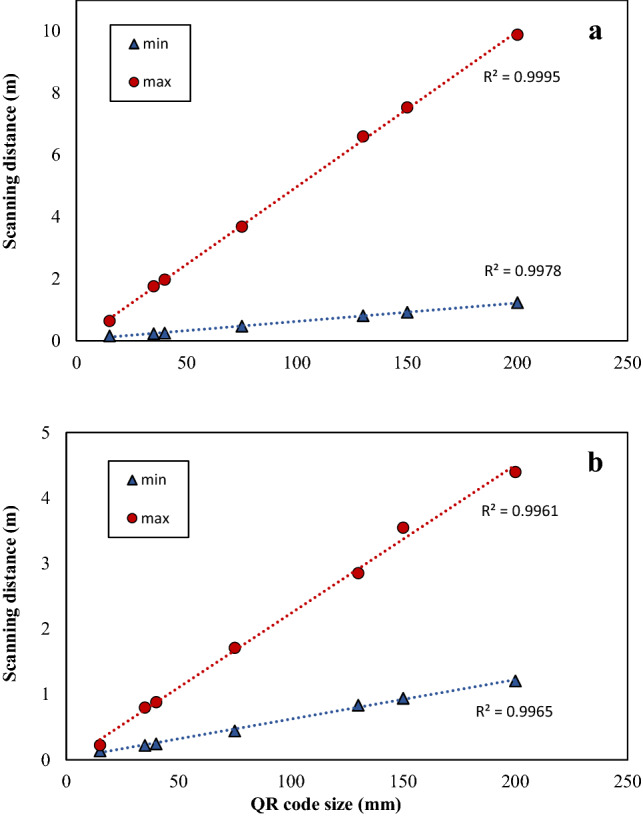
Simple linear regression between minimum and maximum scanning distance (m) and QR code size (mm). Version 3 QR code (V3) with 29 × 29 modules (**a**), Version 11 QR code (V11) with 61 × 61 modules (**b**).

Markers, e.g., QR codes and bar codes, are important elements in AR systems because they represent a valuable tracking method to overlay digital contents on physical and real objects. The capability of the AR device to detect markers and the distance from which they are detectable are aspects that allow us to understand the accessibility of the digital information. For these reasons, the minimum and maximum scanning distances of different sized QR codes using the M400 were evaluated. Figure 2 describes the results obtained from the scanning distance of the V3 (Fig. 2a) and V11 (Fig. 2b) QR codes in relation to their size. As expected, the minimum and maximum scanning distances were directly proportional to the QR code size for both code types. The minimum distance and code dimension ratio for the two types of codes was constant (6.5 on average), as the minimum scanning distance is dictated by the camera framing. A different situation was observed for the maximum scanning distance of the two codes. In fact, the maximum scanning distance was higher for the V3 than the V11 code, with a distance and code dimension ratio of 48.8 and 21.5, respectively. Hence, using the M400 allowed detection of the augmented information from almost 2 m scanning a QR code of 40 × 40 mm size with 29 modules.

These results underline the importance of choosing an appropriate marker type and size to encode the required information. In farms, there are different contexts where specific information are needed to be detected (e.g., form long distance, in a small area, etc.). The scanning distance or the marker size may be influenced by the surrounding context. Furthermore, knowing the maximum scanning distance improves the labor organization. High ambient brightness (full sun) could affect the scanning of markers and thus the augmented information visualization. Although the agricultural context is characterized mainly by outdoor activities where brightness could be a marker-detection-related issue. Previous studies have shown the feasibility to obtain augmented information, by scanning a QR code through the SG camera, during outdoor activities and from the tractor cabin^11^. Moreover, high brightness would mainly affect AR content visualization on devices with the optical see-through systems rather than video see-through systems^30,35^.

Table 2 reports the results of the scanning time where significant differences were recorded among the three QR code sizes considered, which ranged on average between 1.90 s (75 mm) and 2.03 s (35 mm). However, these differences are very low (0.13 s) and do not appear to affect farmer performance.

**Table 2:** Mean, standard deviation (SD) and range of scanning time (s) for three QR code sizes tested (N = 2254).

QR code size (mm)	Scanning time*			
	Mean	SD	Min	Max
35	2.03^a^	± 0.31	1.25	3.42
40	1.98^b^	± 0.29	1.05	3.13
75	1.90^c^	± 0.29	1.00	2.94

^a-c^Values with different letters in the column are significantly different (*P < 0.05).

*Time interval needed to visualize information encoded to the QR code on the smart glasses M400 display.

Moreover, the battery life was monitored during the QR code scanning tests and the videocall tests using both applications (App1, App2). The overall average of 3 h and 28 min was recorded for the scanning tests, whereas 3 h and 50 min and 4 h were recorded for the videocall test, respectively. These values could be a limit if considering the amount of worker hours per day (8 h). In any case, this device should be used for specific and punctual activities carried out on the farm.

The audio and video transmission lag time from the M400 to a laptop was measured to test the quality of a videocall and to evaluate the feasibility of video-remote assistance in real time. The comparison of the results obtained by the two applications (Table 3) showed that the audio transmission was generally less than 0.5 s delay, and no significant difference was observed compared with the transmission quality (Tq). This delay seems to not influence the on-field assistance quality, since delays ranging from 0.4 to 0.15 s are considered acceptable values for high-quality real-time audio transmission^36^.

**Table 3:** Lag time (s) for audio and video transmission from M400 to laptop, using Vuzix Remote Assist (App1) and Brochesia B View (App2) applications. Two transmission qualities (Tq) were tested.

App	Tq	Audio (s)	SD	Video (s)	SD
1	VGA	0.55^ab^	± 0.60	0.55^b^	± 0.51
	HD	0.15^b^	± 0.37	0.90^ab^	± 0.64
2	VGA	0.65^a^	± 0.49	1.25^a^	± 0.72
	HD	0.40^ab^	± 0.50	0.95^ab^	± 0.69

Values with different letters in the column are significantly different (*P* < 0.05).

Considering the lag time for the video transmission, a 0.9 s delay on average was observed for both applications. Moreover, a significant difference of 0.7 s of delay between App1 and App2 was observed within the lower Tq. The results obtained were lower than 3 s on average observed by Muensterer et al.^32^, thus the video conferencing quality of SG may be considered adequate to connect agricultural operators and technicians. Furthermore, the average delay observed in video transmission does not represent a limiting factor since packet loss is more influential on video quality transmission, which was not observed in our tests^37^.

In general, the audio–video lag time was affected by the internet connection. From this perspective, it is important for the farm to have an adequate internet connection (20–30 Mbps) to support the data transfer and video streaming from the SG to a central or remote computer and minimize the delay during a remote videocall.

**Figure 3:**
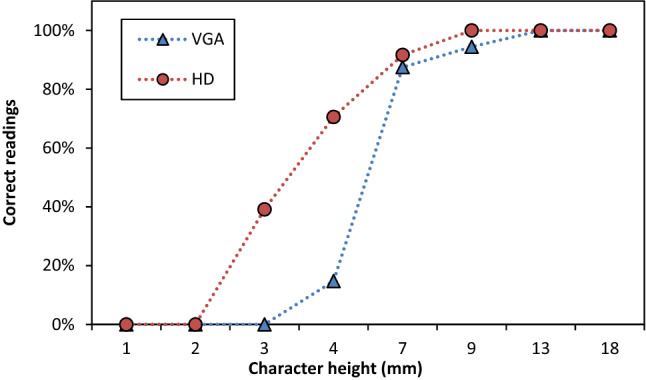
Percentage of correct characters of the Snellen chart read by an operator on the laptop’s screen and transmitted by SG during a videocall. Two resolutions were used: VGA -▲- (640 × 480 pixels) and HD -●- (1280 × 720 pixels). The distance between the smart glasses camera and the chart was 0.5 m.

Figure 3 shows the results obtained from the visual acuity test using the standard Snellen chart. This test allows us to verify the level of content detail transmitted by the SG, which is important when farmers share information in remote assistance with an expert, technician, or another farm operator. The results underlined that a 13 mm character transmitted by the M400, from 0.5 m, on the laptop screen can be read by the receiving operator in every test. In the HD resolution, 9 mm characters were always recognized (100% of characters correctly recognized). On the other hand, 94% of the 9 mm characters were correctly detected at a lower resolution. In addition, it is possible to consider the 7 mm character the lowest printed size easily readable with both resolutions (HD and VGA) with 92% and 88% of the character recognized, respectively. With the 4 mm character size, the gap between the two resolutions increases drastically. In fact, 70% of characters were detected with the HD resolution, in contrast to 15% with the lower resolution. Hance, a farmer with the M400 could share his point of view with a high level of detail with an expert elsewhere, considering that the printed character size is recognizable depending on the videocall resolution.

The transmission results obtained in this study, both in terms of lag time and quality of the video sheering, were found to be appropriate to carry out maintenance activities in milking machine equipment. In fact, small details of the components (milking tubes, claws, liners, valves, etc.) were easily distinguishable from the remote technician.

The interactive system of the M400 includes three buttons, a touchpad, and a vocal interaction (voice commands/control). Considering the voice commands, several trials were carried out to evaluate the capability of the M400 system to respond to the voice action commands enabled at different levels of noise. Studying an environment with soft noise (60–68 dB)^38^ was observed that all the speech commands tested were recognized by the M400 that made the specific action requested (Table 4). A similar situation was observed with a noise level of 70 dB, where for all operators, 89% of the commands were recognized on average. At the 75 dB level of noise, an operator effect was observed. In fact, it was detected that three operators had a mean recognition rate of 79%, while the last operator had a recognition rate of just 5%. Therefore, 70–75 dB represents the border points of speech command detection for the Vuzix M400, considering that noises greater than or equal to 80 dB do not allow the use of the voice control function. This is an important function that, when implemented into the SG, allows the device to be managed completely hands-free. Regardless, in agriculture, the environmental noise can have different levels depending on the activities carried out.

**Table 4:** Action commands correctly recognized (%) by the Vuzix M400 voice control function at different noise levels (dB) and for different operators.

Operator	65 dB (%)	70 dB (%)	75 dB (%)	80 dB (%)	85 dB (%)
1	100	89	92	3	0
2	100	95	65	0	0
3	97	86	81	0	0
4	100	84	5	0	0
Average	99	89	61	1	0

Despite Depczynski et al.^39^ reported that only a few farm conditions stay below the threshold level of 70–75 dB observed for the M400, the noise levels in specific farm situations were measured. Through the sound level meter, the noise related to some agricultural and livestock activities (e.g., use of tractor or milking machine) was monitored to explore in which cases the voice control function of M400 could be effectively used. The results showed that M400 voice control could operate in a cabin tractor (73 kW power), in the milk room and in the milking parlor (Table 5). In fact, the most frequent level of noise measured in the milk room and milking parlor was between 68.7 and 75.3 dB. Moreover, the levels over the threshold detected in these rooms (maximum levels) regarded different sources of sound, e.g., the entry of the animals in the milking parlor, the opening and closing of the gates, and the animals themselves. In contrast, the speech commands were difficult to use when the operator was inside the engine room because the minimum level of decibel recorded was 93.7, which is over the threshold measured for M400. Another agricultural working situation was monitored, involving tractor driving. Two situations were checked: a tractor with a cabin that was more isolated from external noise and a tractor without a cab. The minimum level of decibel recorded allowed us to interact with the M400 by voice, and was recorded in a no working condition or when the tractor engines were running but idling. When the tractors were set in motion, a level of noise over the acceptable threshold was found, as attested by the mode and maximum values recorded for both situations.

**Table 5:** Level of noise in decibel (dB) recorded in different livestock and agricultural working situations/places with the sound level meter.

	Engine room outside	Engine room	Milk room	Milking parlor	Milking parlor 2	Cabin tractor (73 kW)	Tractor without cab (41 kW)
Min	80.8	93.7	67.3	68.6	68.7	58.5	73.6
Max	85.1	98.2	82.2	89.8	90.5	90.7	91.3
Mode	82.0	96.8	68.7	75.3	74.9	78.8	86.3
Average	82.2	96.7	71.4	76.6	76.5	76.3	84.0

*In all scenarios the machines were in operation.

### Milking machine assistance scenario

To verify the proper operation of the milking machine, it is periodically necessary to check the system by certified operators. The ISO 6690:2007 standard specifies mechanical tests for the milking machine to verify installation compliance and components. In this work, a specific milking machine checking process was considered and made available in AR. In particular, the measurement process steps of pulsation characteristics were built on the Brochesia® portal in the workflow section (B Step). The workflow was developed as a sequence of step-by-step instructions that the operator must follow while wearing SG.

Pulsation is the cyclic opening and closing of the liner^34^. The pulsation cycle is composed of 4 phases: liner opening (phase a); liner open (phase b: milking); liner closing (phase c); liner close (phase d: rest). The pulsation characteristics affect milking performance and teat condition^40,41^. Hence, checking the pulsation characteristics as well as the milking system and components is needed at least once or twice a year. The certified operator to verify the system compliance must use specific measuring instruments and follow several procedures depending on the test to be performed, e.g., connect the sensors (flowmeter, pulsograph), disconnect some components of the milking machine (vacuum controller). Commonly, operators are supported by a paper guide or paper checklist to ensure that all test procedures are performed correctly. In this context, it appears that an augmented reality procedure supported by icons and images (visual information procedures) might be an important element in learning and performing this type of test. In addition, an SG-specific maintenance application for these mechanical tests is even more important, considering that during maintenance procedures, the operators need to make various movements and use their hands frequently.

**Figure 4:**
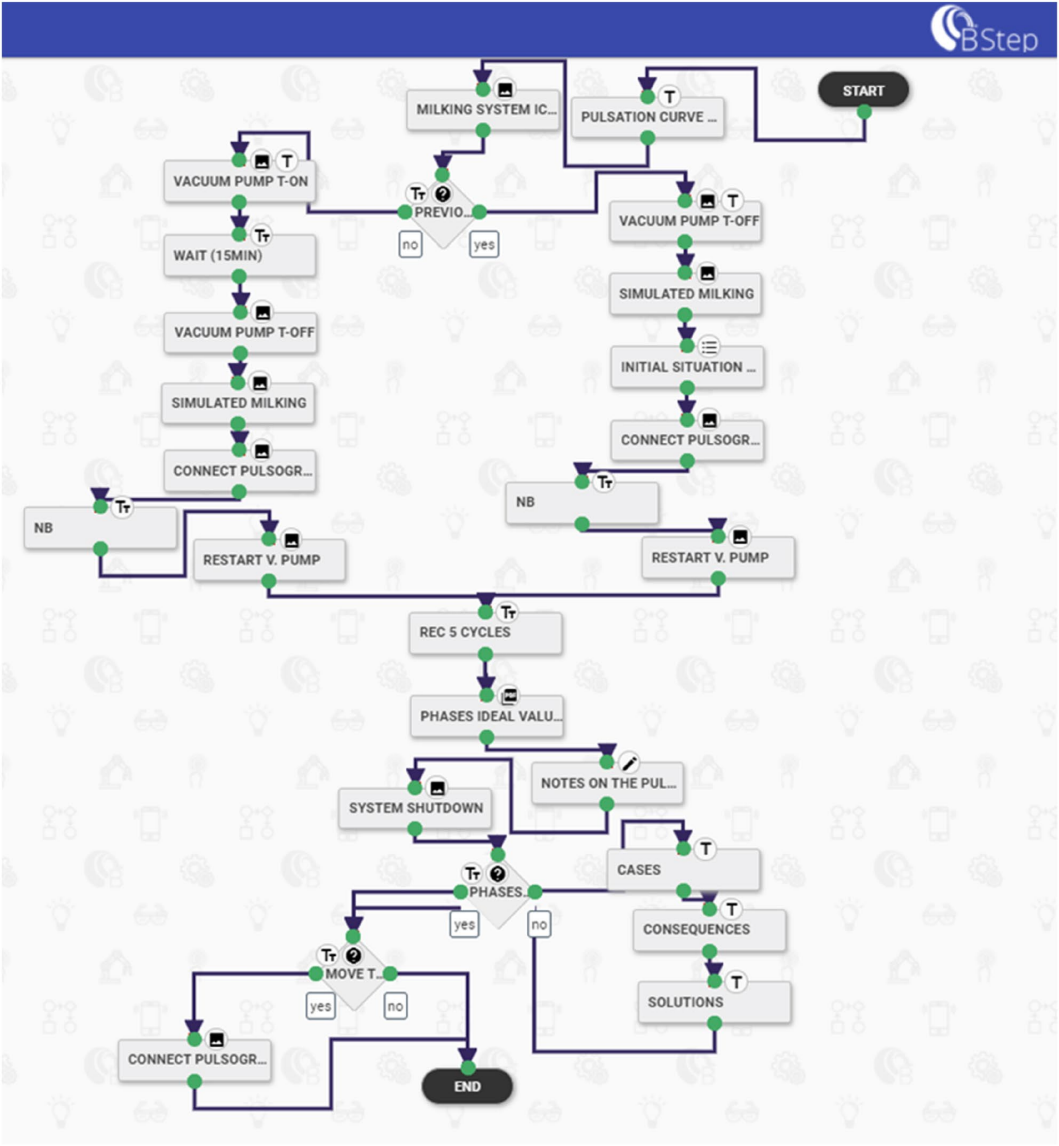
Mechanical milking installations tests: workflow scheme built with the BStep application software describing the steps of the pulsation characteristics test.

The test for pulsation characteristics consists of several steps and is related to the previous tests carried out on the milking machine. Three steps are fundamental: the connection of the pulsograph to the machine, the recording of five complete pulsation cycles and the evaluation of the data recorded for each milking unit, i.e., length of the a, b, c, and d phases per pulsation cycle. In Fig. 4, the workflow scheme of the pulsation characteristics process developed on the BStep application portal is reported, following the ISO 6690:2007 standard.

**Figure 5:**
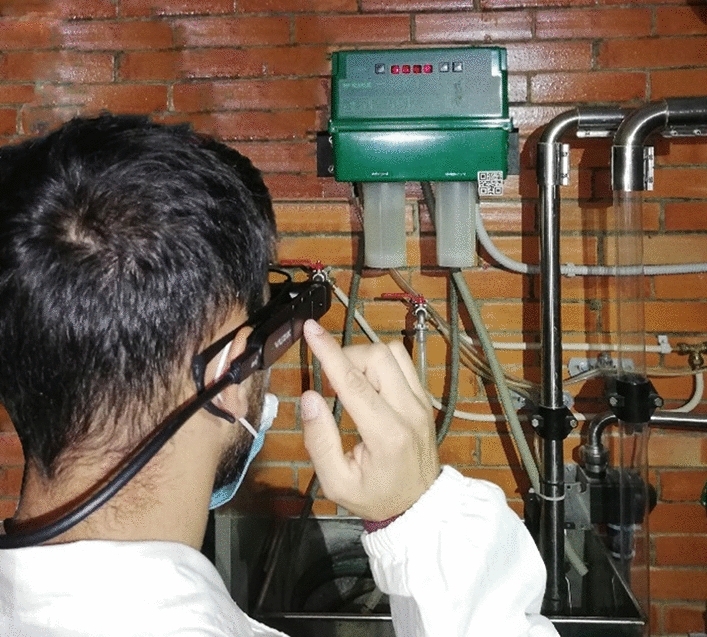
Operator with SG while accessing the maintenance augmented instructions through the QR code (40 mm), placed on the milking machine management control unit (green box).

The workflow of the pulsation characteristics test was available from a 40 × 40 mm QR code with 29 modules placed on the milking machine (Fig. 5). The QR code represented the marker that the operator needed to scan with the SG to obtain the augmented information. As observed in the previous tests, this type of QR code can be scanned quickly from a distance of 2 m using the M400. In addition, although different sources of noise were found in the milking parlor (animal noises, pulsators, vacuum regulator valve, etc.), where the maintenance scenario was contextualized, the noise levels most frequently recorded did not affect the use of SG by voice commands (Table 5). Therefore, the operator was able to proceed with the milking machine and components inspection while having his hands completely free.

**Figure 6:**
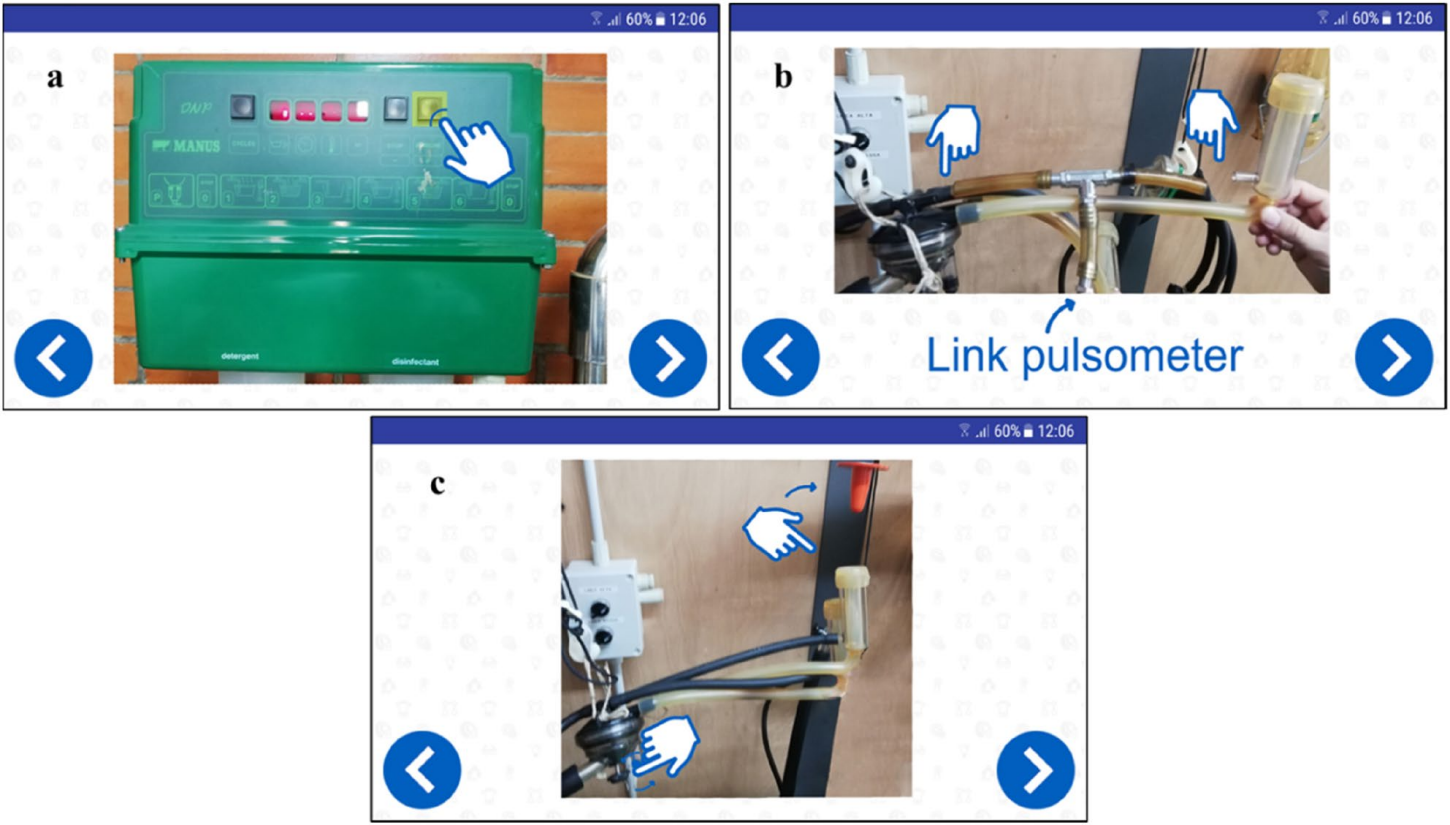
Example of augmented information visualized on the smart glasses M400 during the mechanical milking machine test through the BStep app. (**a**) Indications for vacuum pump ignition, (**b**) indication for the sensor attachment (T connection); (**c**) setting the milking machine in simulated milking (moving the claw valve and closing the liner with the specific plug).

The first augmented instruction provided to the operator was the question: *Is this the first test performed on the milking machine*? which implies if other instruments meter and configurations are in place or not. Interacting with the workflow by the touchpad of the M400, the operator can choose two different ways (Fig. 4). Nevertheless, some preliminary steps before checking the pulsation were needed: in one case, e.g., stop the vacuum pump and set the system in simulated milking, mount vacuum regulator, disconnect the flowmeter (if previously used); in another case, turn on the vacuum pump (Fig. 6a), wait fifteen minutes, set the system in simulated milking, etc., as shown in Fig. 6c. Then, attaching the sensors of the pulsograph with the T-piece connection was requested and the right way was explained through the augmented instructions (Fig. 6b). Afterward, recording a minimum of 5 pulsation cycles with the test equipment to obtain reliable data was indicated. The recommended values regarding the length of each phase (a, b, c, d) were visible on M400 to support the operator during the evaluation of the data recorded in all milking units. When anomalous pulsation curves were observed, the operator was able to view tips and suggestions to solve the problem and restore the pulsation system.

Overall, the results obtained confirmed the suitability of the device tested for milking machine maintenance. The aspects characterizing the agricultural environment such as remote location, limited or difficult access routes, and reduced number of specialized maintenance workers per farm, are the main driving factors for using AR and SG as a support tool for specialized remote assistance. Additionally, the agricultural environment is remarkably heterogeneous and different from other workplace (i.e., industry sector) where different operating situations (field crop management, greenhouse, barn animals, grazing animals, etc.) and different machinery (tractors, implements, milking equipment, etc.) can coexist. The milking machine is one of the farm machines that require specific maintenance by skilled technicians. As indicated by ISO 6690:2007, several mechanical components (vacuum pump, milking units, pulsators, etc.) and operative parameters during milking must be checked periodically for an efficient milking operation^42^. Failures during milking of such equipment can cause economic losses due to a decreasing milk yield. Therefore, prompt and precise maintenance is necessary both in emergency situations and in scheduled checks.

## Conclusions

In this work, the performance of specific smart glasses for augmented reality (Vuzix M400) was evaluated from an agricultural point of view. Thus, considering some features of the device (QR code scanner, AR image and video quality transmission) and the possibility of installing applications, a step-by-step maintenance scenario in augmented reality was developed to support technicians during the mechanical tests of the milking machines.

The tests underlined that the SG has a natural aptitude to be employed in the field of agricultural assistance. Specifically, the features of the SG adopted (AR precise content visualization, video transmission, voice control) could improve the operators’ performance in the maintenance of on farms machines (tractors, milking machines, food processing equipment, etc.). In fact, the results showed the capability of SG to reach the AR information from a long distance (through a QR code) in a short time interval and to transmit audio and video with a high level of detail, allowing discrimination of small objects during remote assistance with reduced delay. Moreover, the tests allowed us to emphasize that this device can be managed completely hands-free with the voice control function but only in situations with a maximum noise level of 70–75 dB, depending on the voice tone of the user.

In relation to this application context, a simulated maintenance scenario was built with an external service, representing an example of an AR remote assistance application in the agricultural context. The proposed maintenance scenario underlines the potential that AR could have in the agricultural sector to assist and support both farmers and technicians to solve problems while working on simultaneously visualizing different information on the SG. In relation to AR potentiality for the agricultural sector, more complex but not complicated, applications or assistance scenarios should be developed considering the farmers’ needs, also thanks to the implementation of cloud computing where the AR instruction for the maintenance of agricultural machines can be stored.

Overall, this study highlighted how video see-through SG can be used for agricultural machinery maintenance by providing digital guidance to the operator. Although the type of visualization system may be found in other handheld devices (smartphones and tablets), these devices do not leave the operator's hands free to simultaneously perform the tasks while receiving augmented instructions. Besides the highlighted attitudes of the SG tested, some critical aspects were found in the field of view (larger display size would help AR contents visualization), in the battery life (at least two extended power banks are suggested) and in the wearability (long time usage may result uncomfortable). Important opportunities will be provided by mixed reality (MR) devices (e.g., Microsoft Hololens2) equipped with Time of flight (ToF), eye and hand tracking sensors, spatial mapping and higher computational power, in order to accomplish complex tasks in the agricultural domain. However, MR devices, such as Hololens2 have higher costs, and outdoor activities may be compromised by direct sunlight. Anyway, future studies are needed to understand whether MR devices are suitable in agricultural use-case scenarios. Moreover, the intention to use of this kind of technology from the agricultural stakeholders should be assessed using proper evaluation models in future studies.

## Data Availability

The datasets generated during the current study are available from the corresponding author on reasonable request.
